# Transient effects of transfusion and feeding advances (volumetric and caloric) on necrotizing enterocolitis development: A case-crossover study

**DOI:** 10.1371/journal.pone.0179724

**Published:** 2017-06-20

**Authors:** Vi T. Le, Mark A. Klebanoff, Maria M. Talavera, Jonathan L. Slaughter

**Affiliations:** 1 Center for Perinatal Research, The Research Institute at Nationwide Children’s Hospital, Columbus, OH, United States of America; 2 Department of Pediatrics, The Ohio State University, Columbus, OH, United States of America; Univesity of Iowa, UNITED STATES

## Abstract

**Objective:**

To evaluate the short-term effects of feed fortification, feed volume increase, and PRBC transfusion on the odds of developing NEC.

**Study design:**

Case-crossover study of neonatal intensive care infants born at ≤ 32 weeks’ gestation who were admitted to 5 central Ohio intensive care units from January 2012-July 2016 and developed NEC Bell Stage ≥2. Each patient served as their own control, with exposure during the 48-hour period just prior to NEC onset (hazard period) being compared to a preceding 48-hour control period, thus eliminating confounding by patient factors fixed between both intervals. NEC onset was determined by chart review as the earliest occurrence of one of the following within 24 hours of confirmatory x-ray: (1) antibiotic initiation, (2) enteral feeding cessation, (3) physician first notified of abdominal concerns, or (4) abdominal x-ray ordered. Conditional logistic regression compared exposures to feed volume increase, fortification, and PRBC transfusion during the 48-hour period prior to NEC onset to those during a preceding 48-hour control period. Analyses were stratified by gestational age and anemia (defined: hemoglobin ≤ 9.3 g/dL within 7 days of NEC onset).

**Results:**

We included 63 infants with confirmed NEC. Acute exposure to fortification (odds ratio [OR]: 1.67, 95% confidence interval [CI]: 0.61, 4.59), feed volume increase (OR: 0.63, 95% CI: 0.28, 1.38), and PRBC transfusion (OR: 1.80, 95% CI: 0.60, 5.37) was not associated with the onset of NEC. Gestational age and anemia did not significantly modify the associations. Sensitivity testing substituting 24- and 72-hour hazard and control periods produced similar results.

**Conclusion:**

Using a case-crossover design, we did not detect an association between NEC development and feed fortification, feed volume increase, or PRBC transfusion within 48-hours prior to NEC-onset. Replication in a larger set of cases is needed.

## Introduction

Necrotizing enterocolitis (NEC) is a devastating illness in the neonatal intensive care unit (NICU) due to its association with severe morbidities and increased mortality [1, 2]. Despite intensive research efforts, the pathogenesis of NEC remains poorly understood. Symptoms and early radiological features are commonly nonspecific, reflecting the lack of clear, consistent diagnostic criteria [2].

Developing preventive measures for NEC has been difficult with inconclusive and conflicting data regarding the contribution of acute exposures, such as enteral feeding advancement [3] and packed red blood cells (PRBC) transfusions [4, 5]. Exploring these risk factors is important as over 90% of NEC infants received enteral feedings [6] and PRBC transfusions are common among neonates in the NICU. Prior retrospective observational studies have suggested a cautious approach to feeding advancements [7] and PRBC transfusion [4]; however, clinical trials [3, 8] and a prospective study [5] have produced contradictory results. Findings obtained from observational studies were potentially affected by confounding [4, 5] and, especially in database-focused investigations, could have failed to evaluate the temporality between exposures and NEC development. The case-crossover design provides possible solutions to some of the methodological issues in observational studies [9]. With this design, each infant serves as his/her own control, thereby allowing the control of fixed characteristics (such as Apgar scores, gestational age, birth weight, race, and other unmeasured extraneous variables) that could confound the temporal associations. The case-crossover study design is best suited to assess the transient effects of acute exposures on diseases with abrupt onset [9]. The design has been used to determine exercise as a trigger for myocardial infarction [10] and cellular telephone usage as a cause of vehicle collision [11].

The objective of this study was to perform a case-crossover analysis in order to evaluate the temporal associations between the acute exposure to feed fortification, feed volume increase, PRBC transfusion and NEC onset.

## Materials and methods

Data were obtained from the Nationwide Children’s Hospital’s (NCH) Electronic Data Warehouse, an administrative database created through a consortium of 7 Central Ohio maternity hospitals. Services are distributed among 5 level III NICU and 3 level II special care units. All neonatal units at NCH employed standardized feeding protocols that emphasized early feedings and the use of human breast milk [12]. Infants <28 weeks’ gestation were given trophic feeds of 10 mL/kg/day every 6 hours for the first 3 days. If tolerated, feeds increased to every 3 hours on days 4 to 6. On the seventh day of life, feeding volume increased at 20 ml/kg/day until full feeds were achieved at 150 mL/kg/day volume. Feeds were held during transfusions, and fortification and volume advancement were discouraged on the day of transfusion. Human milk feedings were fortified with Similac human milk liquid fortifier using manufacturers’ recommendations. Fortification to 22 kcal/oz began when feeds reached 100 ml/kg/day. Following 24 hours of feeding tolerance, fortification was advanced to 24 kcal/oz.

All infants admitted to NICUs within the system from January 2012-July 2016 diagnosed with NEC (ICD-9 codes: 777.50, 777.51, 777.52, 777.53; ICD-10 codes: P77, P771, P772, P773, or P779) were assessed for eligibility. We included all infants of ≤32 weeks’ gestation and excluded infants with severe congenital or genetic abnormalities. Nationwide Children’s Institutional Review Board approved the protocol (IRB16-00650) and waived the need for informed consent since this was a retrospective analysis of data collected during routine clinical care.

The primary outcome was definite NEC, defined as Bell stage ≥2. A manual medical record review was conducted in order to determine the certainty of NEC staging. We reviewed clinical notes, surgical reports, and abdominal x-ray reports, specifically recording the presence of persistent pneumatosis intestinalis, perforation, portal venous gas, and fixed bowl loops. Spontaneous perforation was considered a different disease entity and was not included in analyses. Data concerning demographics, diagnoses, transfusion, laboratory, and enteral feeding variables were collected.

### Timing of NEC onset

Among confirmed NEC cases, onset was defined as the earliest of the following events within 24 hours of confirmatory x-ray reports: 1) the first notification of abdominal concerns to physicians (first indication of NEC for 51% of cases), 2) order placed for abdominal x-rays (first indication for 36% of cases), 3) enteral feeding cessation (first indication for 10% of cases), or 4) antibiotic initiation (first indication for 3% of cases). This definition was used to verify the timing of PRBC and feeding exposures in the absence of early NEC symptoms and to eliminate the possibility of ascertaining exposures that are secondary to NEC.

### Variables

For each infant, we analyzed procedural and dietary orders on the day of NEC onset and the preceding 7 days in order to collect timing of feed volume increases, fortification, and PRBC transfusion. Hemoglobin and hematocrit lab results were collected in order to determine anemia, defined as a hemoglobin level of ≤9.3 g/dL within 7 days of NEC onset. Anemia was defined by considering hemoglobin thresholds from local transfusion protocol recommendations for infants requiring respiratory support, although individual providers do not have to adhere to those recommendations. Gestational age was characterized into a binary variable, ≤28 and >28 weeks, since younger infants are more likely to receive PRBC transfusions and develop NEC [13].

### Definition of control and hazard periods

Our primary analyses compared each infant's exposure in the immediate 48-hour period just prior NEC onset (hazard period) to a preceding 48-hour control period. Additional analyses tested the robustness of our results by altering the hazard and control period to 24-hours and 72-hours.

### Statistical analysis

Conditional logistic regression, adjusting for the self-matching design, was used to estimate odds ratios (ORs) and 95% confidence intervals (CIs) comparing exposures to feed volume increase, fortification, and PRBC transfusion in the hazard period to those within the control period. We stratified analyses by gestational age and anemia in order to assess potential modifications of ORs. All statistical analyses were conducted in Stata 14 (StataCorp LP., College Station, Texas).

## Results

**Fig 1:**
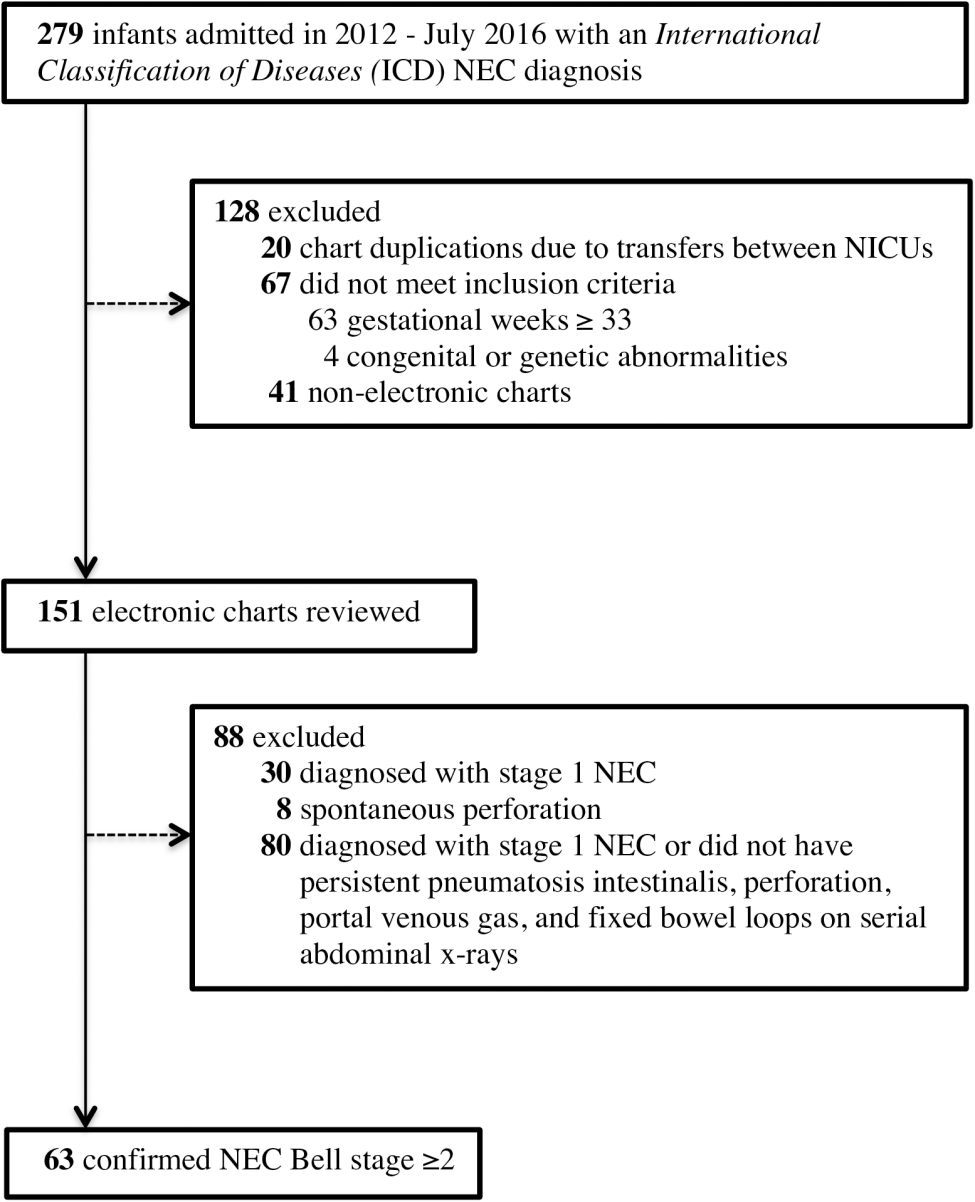
Flowchart of necrotizing enterocolitis (NEC) study cohort.

Of the 279 NICU infants admitted between January 2012 and July 2016 with an ICD diagnosis for NEC, 151 infants from 5 hospitals met our inclusion and exclusion criteria and were subjected to a chart review (Fig 1). Among infants whose medical records were reviewed, 63 (41.7%) of those infants were confirmed with Bell stage ≥2 using results from abdominal x-rays and reviews from clinical progress notes. Antibiotic initiation, enteral feeding cessation, abdominal x-rays orders, and first notification of abdominal concerns to physicians all occurred within 24 hours of confirmatory x-rays reports in 92.1% of confirmed Bell stage ≥2 cases. The majority of infants were ≤28 gestational weeks (Table 1). In the week prior to NEC onset, 52 (82.5%) of cases were fed breast milk. Among the 26 NEC infants who received fortification in the week prior to NEC onset, 15 (57.7%) received fortification through the addition of human milk fortifiers while 11 (42.3%) received fortification through the addition of formulas (i.e., Similac Special Care, Similac Expert Care Alimentum, or Neosure).

**Table 1:** Characteristics of confirmed NEC Bell stage ≥2 (N = 63).

Characteristics	n (%)
**Birth gestation, weeks**	
** < 24**	8 (12.7)
** 24–26**	23 (36.5)
** 27–28**	11 (17.5)
** 29–32**	21 (33.3)
**Female**	32 (50.8)
**Race, n (%)**	
** White**	36 (57.1)
** Black/African American**	18 (28.5)
** Asian**	3 (4.8)
** Other**	3 (4.8)
** Unknown**	3 (4.8)
**Ethnicity**	
** Non-Hispanic**	53 (84.1)
** Hispanic**	6 (9.5)
** Unknown**	4 (6.4)
**Singleton Birth**	51 (81)
**Birth weight, grams, median (IQR)**	810 (650–1155)
**5-minute Apgar Score, median (IQR)**	7 (4–8)
**Clinical Characteristics**	
** NEC onset, day, median (IQR)**	23 (9–39)
** Received fortification 7 days prior to NEC**	26 (41.3)
** Received feed volume increase 7 days prior to NEC**	54 (85.8)
** Received PRBC transfusion 7 days prior to NEC**	21 (33.3)
** Surgical NEC**	27 (42.9)
** Death**	23 (36.5)

Abbreviations: NEC, necrotizing enterocolitis; IQR, interquartile range; PRBC, packed red blood cells

### Effects of feeding advancement

Fortification within 48 hours of NEC onset was not associated with NEC (odds ratio [OR]: 1.67, 95% confidence interval [CI]: 0.61, 4.59) (Table 2). Higher odds for fortification were found among those born at ≤28 gestational weeks (OR: 2.00, 95% CI: 0.60, 6.64), compared to those with >28 weeks (OR: 1, 95% CI: 0.14, 7.10); however, results were limited by the contribution of 12 and 4 discordant pairs, respectively (Table 3). Similar results were obtained with the use of 24-hour and 72-hour hazard and control periods (Table 4).

**Table 2:** Odds ratio (OR) for confirmed NEC in infants exposed to feeding advancement and PRBC transfusion 48 hours prior to NEC onset.

	A	B	C	D	OR (95% CI) ^a^
**Feed fortification**	5	10	6	11	1.67 (0.61, 4.59)
**Increase in feeding volume**	19	10	16	9	0.63 (0.28, 1.38)
**PRBC transfusion**	4	9	5	20	1.80 (0.60, 5.37)

Abbreviations: NEC, necrotizing enterocolitis; PRBC, packed red blood cells

A: Number of concordant pairs exposed in both hazard and control period (n_11_)

B: Number of discordant pairs exposed in hazard period, unexposed in control period (n_10_)

C: Number of discordant pairs unexposed in hazard period, exposed in control period (n_01_)

D: Number of concordant pairs unexposed in both hazard and control period (n_00_)

^a^ OR defined as B/C or n_10_/ n_01_

**Table 3:** Odds ratio (OR) for confirmed NEC in infants exposed to feeding advancements and PRBC transfusion 48 hours prior to NEC onset according to gestational weeks and anemia.

		A	B	C	D	OR (95% CI) ^a^	P value ^b^
**Feed fortification**	Gestational weeks						
	≤28	2	8	4	8	2 (0.60, 6.64)	0.56
	>28	3	2	2	3	1 (0.14, 7.10)	
**Feeding volume increase**	≤28	7	7	11	8	0.64 (0.25, 1.64)	0.95
	>28	12	3	5	1	0.60 (0.14, 2.51)	
**PRBC transfusion**	≤28	4	9	5	14	1.80 (0.60, 5.37)	-
	>28	-	-	-	6	-	
	Anemia^c^						
	Anemia	1	6	1	6	6 (0.72, 49.8)	0.19
	No Anemia	3	3	3	14	1 (0.20, 4.95)	

Abbreviations: NEC, necrotizing enterocolitis; PRBC, packed red blood cells

A: Number of concordant pairs exposed in both hazard and control period (n_11_)

B: Number of discordant pairs exposed in hazard period, unexposed in control period (n_10_)

C: Number of discordant pairs unexposed in hazard period, exposed in control period (n_01_)

D: Number of concordant pairs unexposed in both hazard and control period (n_00_)

^a^ OR defined as B/C or n_10_/ n_01_

^b^ X^2^ test with one degree of freedom was used to compare ORs within subgroups

^c^ Hemoglobin level of ≤9.3 g/dL within 7 days of NEC onset

**Table 4:** Sensitivity analyses showing odds ratio (OR) for confirmed NEC in infants exposed to feeding advancements and PRBC transfusion 24 and 72 hours prior to NEC onset.

	Hazard Period	A	B	C	D	OR (95% CI) ^a^
**Feed fortification**	24 hours	2	7	6	17	1.17 (0.39, 3.47)
	72 hours	6	12	8	6	1.50 (0.61, 3.67)
**Feeding volume increase**	24 hours	9	7	13	25	0.54 (0.21, 1.35)
	72 hours	32	8	11	3	0.73 (0.29, 1.81)
**PRBC transfusion**	24 hours	0	7	6	25	1.17 (0.39, 3.47)
	72 hours	3	12	5	18	2.40 (0.85, 6.81)

Abbreviations: NEC, necrotizing enterocolitis; PRBC, packed red blood cells

A: Number of concordant pairs exposed in both hazard and control period (n_11_)

B: Number of discordant pairs exposed in hazard period, unexposed in control period (n_10_)

C: Number of discordant pairs unexposed in hazard period, exposed in control period (n_01_)

D: Number of concordant pairs unexposed in both hazard and control period (n_00_)

^a^ OR defined as B/C or n_10_/ n_01_

Volumetric increase within 48-hours of NEC onset was not associated with NEC (OR: 0.63, 95% CI: 0.28, 1.38) (Table 2). Gestational age did not significantly modify the relationship between the 48-hour control and hazard period, p = 0.95 (Table 3). Results did not differ with the use of 24-hour and 72-hour hazard and control periods (Table 4).

### Effects of PRBC transfusion

Among the 18 cases who received a PRBC transfusion with the 48-hour period prior to onset, 7 cases died and 10 cases required surgery. PRBC transfusion within the 48-hour period prior to onset was not associated with NEC (OR: 1.80, 95% CI: 0.60, 5.37), although the OR was based on 14 discordant pairs (Table 2). Anemia did not significantly modify the relationship between PRBC transfusion and NEC. The effect of PRBC transfusion on NEC development was greater among those with anemia (OR: 6, 95% CI: 0.72, 49.8), compared to those without anemia (OR: 1, 95% CI: 0.20, 4.95), but the difference was not statistically significant (Table 3). Results were consistent with the use of 24-hour and 72-hour hazard and control periods (Table 4).

## Discussion

In this case-crossover analysis, we did not find an association between the transient effects of enteral feeding advancements and PRBC transfusion with NEC development. Gestational age did not significantly modify the relationships between feeding advancements and NEC. Moreover, the relationship between PRBC transfusions and NEC development was not significantly modified by anemia.

Cautious advancement of enteral feeds is often recommended in order to reduce NEC occurrence. However, a slow feeding regime could emphasize prolonged usage of total parenteral nutrition and lead to complications that increase morbidity and mortality [8, 14, 15]. We failed to find an association between the short-term effects of fortification and NEC development. Similarly, a Cochrane review of 11 randomized and quasi-randomized trials concluded no significant differences in NEC development between infants who received fortified breast milk and those who received unfortified breast milk [3]. Moreover, we did not find an association between the short-term effects of feeding volume increase and NEC development. This was in contrast to the observational studies suggesting an association between rapid feeding advancement and NEC [7, 16]. However, results aligned with a Cochrane review of 9 randomized control trials that concluded the daily feed advancements at 30 to 40 mL/kg increments, compared to increments of 16 to 24 ml/kg, did not increase the risk of NEC [8]. Albeit non-significant, feeding volume increase appeared to be protective of NEC development. It is plausible that symptoms of subclinical NEC may have led to cautious advancement of feeds or that increases in feeding volume may signal a rapid feeding regime (thus increasing the ability to reach full feeds faster) and ultimately resulting in less sepsis-related complications [8, 15]. Our findings indicate that the acute exposure to enteral feeds increase was not associated with NEC and raises the potential concern for unintended detrimental consequences that may result from conservative feeding practices, particularly since optimal nutrition mitigates the risks of adverse growth and neurodevelopmental outcomes [15].

Studies exploring the relationship between PRBC transfusions and NEC have produced conflicting results. Our findings were in contrast to the meta-analysis of 12 retrospective observational studies that concluded an association between PRBC transfusions and NEC (unadjusted OR: 3.91, 95% CI: 2.97, 5.14; adjusted OR: 2.01, 95% CI: 1.61, 2.50) [4]. However, our results were consistent with a more recent meta-analysis of 23 observational studies (one of which included a prospective observational analysis [5]) that did not conclude an association (OR: 1.13 95% CI: 0.99, 1.29) [17]. Specifically, Hay et al concluded “low” to “very low” confidence of a true relationship between transfusion and NEC, based on the Grading of Recommendations Assessment, Development, and Evaluation (GRADE) system. The classification was attributable to the bias in underlying studies, stemming from unadjusted baseline characteristics, confounding by indication, inconsistent and nonspecific NEC definitions, and the failure to assess temporarily between PRBC transfusions and early NEC onset [5]. By employing a case-crossover design and conducting a retrospective chart review, the bias of previous observational studies may be mitigated from the results of this study. Our study design allowed us to control for fixed baseline characteristics, verify the certainty of a NEC Bell stage ≥2, and determine the timing of the early pathophysiological signs of NEC in relation to PRBC transfusion.

Several studies have suggested anemia as a risk factor in NEC development, possibility through the induction of mucosal injuries relevant to NEC susceptibility [18–20]. Ho et al found a significant association between PRBC transfusion and mucosal inflammation, with higher levels of mucosal inflammation found among infants with lower pre-transfused hematocrit levels [19]. Similarly, findings from retrospective analyses [18, 21] and meta-analysis of 3 clinical trials [17, 22] have suggested an inverse relationship between pre-transfused hematocrit levels and the risk of NEC. In a prospective observational analysis, Patel et al demonstrated an increased risk of NEC among very low birth weight infants with severe anemia compared to those without severe anemia (adjusted hazard ratio: 5.99, 95% CI: 2.00–18.0) [5]. The definition of anemia we utilized, reflecting local transfusion practice at NCH, was less severe than that used by Patel et al. This difference may have moved our results toward the null if prevention of severe anemia does in fact prevent NEC.”

Our results showed the effect of PRBC transfusion on NEC onset was greater among those with anemia compared to those without anemia, albeit not significantly so. By design, our case-crossover investigation only evaluated short-term exposures in patients with a confirmed NEC diagnosis. Thus, we were unable to evaluate an association between anemia and NEC, both because anemia is a longer-term exposure and such an evaluation would require a cohort containing at-risk patients that both did and did not develop NEC. Therefore, we can only speculate on our finding of elevated, but non-significant, odds of NEC in patients with anemia who were transfused within 48-hours of NEC onset. It is possible that anemia itself is a causal risk factor for NEC [23] and our finding of a potential interaction effect may be merely because transfusion is positively associated with both anemia and NEC. Another potential explanation might be that the non-anemic patients who received transfusion were less sick and thus, without potential yet unknown risk factors that might predispose to NEC. Our findings highlight the need for further assessments concerning the interaction between anemia and PRBC transfusions in NEC susceptibility. Notably, our cohort included 10 infants who received a PRBC transfusion in the absence of anemia, per our definition. Although these infants were transfused at a higher hemoglobin threshold, hematocrit and hemoglobin thresholds for anemia vary based on clinical characteristics and decisions concerning PRBC transfusion can be influence by oxygen requirements and the need for respiratory support [24]–factors that may affect NEC susceptibility or suggest the presence of prodromal indicators of NEC.

Our study has several limitations. Due to the small number of NEC patients, we had insufficient power to detect statistical differences in our analyses. This was particularly true when comparing anemic and non-anemic infants, as well as those born at ≤28 and >28 gestational weeks, who received a transfusion. Moreover, we were unable to stratify our analyses based on the types of feed. Even though we failed to detect statistical significance, the higher odds associated with PRBC transfusion and feed fortification may signal an important relationship. Our results remained consistent when we employed various hazard and control period definitions. Nevertheless, this is the first study, to our knowledge, that used the case-crossover design to study the short-term effects of PRBC transfusion and feeding advancement in NEC development. Thus, our study design mitigated bias affected by unadjusted differences in baseline characteristics, a potential source of bias in previous observational studies [4, 17]. Reverse causation was possible if symptoms of subclinical NEC may have led to transfusion or cautious advancement of feeds. We were able to mitigate this possibility by conducting a manual chart review and classifying NEC at time of onset, as opposed to time of disease confirmation. Specifically, we were able to exclude PRBC transfusions and feed advancements that were secondary to prodromal NEC and identify exposures predating the preclinical signs of NEC. However, it is plausible that our stringent definition of NEC might have biased results toward the null. Lastly, generalizability of these results may be limited. Findings were from a single-center analysis, and protocols surrounding feeding practices and PRBC transfusions may vary. Due to study design, our findings can only estimate the short-term effects of PRBC transfusions and feeding advances on NEC susceptibility, and we were unable to examine the association between anemia and NEC since anemia can be a chronic condition.

## Conclusions

The case-crossover design, used to assess the transient effects of acute exposures on outcomes, can reduce confounding from clinical characteristics through its use of self-matching. By using this rigorous study design, we did not detect an association between the short-term effects of feed fortification, feed volume increase, PRBC transfusion and NEC development. Further studies are needed to examine the impact of conservative feeding practices and the interaction between anemia and PRBC transfusion in NEC susceptibility. Additional case-crossover studies employing the same protocols would be useful in increasing the power to detect true differences and the precision of our results.
